# A two-step approach for fluidized bed granulation in pharmaceutical processing: Assessing different models for design and control

**DOI:** 10.1371/journal.pone.0180209

**Published:** 2017-06-29

**Authors:** Liangshan Ming, Zhe Li, Fei Wu, Ruofei Du, Yi Feng

**Affiliations:** 1 Engineering Research Center of Modern Preparation of TCM of Ministry of Education, Shanghai University of Traditional Chinese Medicine, Shanghai, China; 2 College of Chinese Materia Medica, Shanghai University of Traditional Chinese Medicine, Shanghai, China; Universita degli Studi della Tuscia, ITALY

## Abstract

Various modeling techniques were used to understand fluidized bed granulation using a two-step approach. First, Plackett-Burman design (PBD) was used to identify the high-risk factors. Then, Box-Behnken design (BBD) was used to analyze and optimize those high-risk factors. The relationship between the high-risk input variables (inlet air temperature X_1_, binder solution rate X_3_, and binder-to-powder ratio X_5_) and quality attributes (flowability Y_1_, temperature Y_2_, moisture content Y_3_, aggregation index Y_4_, and compactability Y_5_) of the process was investigated using response surface model (RSM), partial least squares method (PLS) and artificial neural network of multilayer perceptron (MLP). The morphological study of the granules was also investigated using a scanning electron microscope. The results showed that X_1_, X_3_, and X_5_ significantly affected the properties of granule. The RSM, PLS and MLP models were found to be useful statistical analysis tools for a better mechanistic understanding of granulation. The statistical analysis results showed that the RSM model had a better ability to fit the quality attributes of granules compared to the PLS and MLP models. Understanding the effect of process parameters on granule properties provides the basis for modulating the granulation parameters and optimizing the product performance at the early development stage of pharmaceutical products.

## Introduction

Granulation is defined as a process for size enlargement. In this process, small powder particles are brought into contact with each other to form a semi-permanent aggregation in which the original particles can still be distinguished [1]. Unlike high-shear granulation and screw granulation, in which the wet granules are transferred to a drying unit, fluidized bed granulation is a one-step continuous operation including dry mixing, wetting, and drying. Therefore, it reduces the number of unit processes, thus improving the production efficiency, reducing the cost, and satisfying the cGMP requirements [2]. Fluidized bed granulation has many advantages such as simple process and cost saving. Therefore, it is used widely in the chemical and pharmaceutical industries, as well as in agriculture, but its applications are still guided mostly by the empirical trial-and-error methods [3]. To better understand fluidized bed granulation and achieve the desired target product profile, a quality by design (QbD) framework in which the quality is a built-in property rather than a measured test of the final product, should be emphasized and carried out. To provide a mechanistic basis for process understanding, a design of experiment (DoE) approach is recommended. In this approach, a design space with the desired characteristics is established, and the high-risk variables critical to the final product quality are identified [4]. Granulation parameters should be properly controlled in order to obtain high-quality granules. During fluidized bed granulation, the output is highly dependent upon the energy and binder input, in that higher inlet air temperature and velocity and a lower binder addition rate result in spray-drying rather than agglomeration. Conversely, with lower inlet air temperature and velocity and a higher binder addition rate, there is a transition from pneumatic delivery to de-fluidization. Improperly setting parameters, either spray-drying or coating, may result in uncontrollable granulation behavior. Therefore, understanding the operating mechanisms is a prerequisite for reliably obtaining proper quality granules in granulation.

In fluidized bed granulation, the effects of binder properties and excipients on granulation and the properties of granulation product have been studied [5, 6]. Various other operational conditions and properties of granules have also been investigated by multilinear regression analysis [7]. The granulation of cohesive Geldart group C powder was successfully carried out using a Mini-Glatt fluidized bed using precoated nanosilica [8]. The common mechanisms for the aggregation of powders were wetting and nucleation, consolidation and growth, and breakage and attrition [9]. Moreover, the development of in-line process analytical technology (PAT) has made it possible to understand the process and further elucidate the potential mechanisms in fluidized bed granulation using near-infrared (NIR) spectroscopy [10], spatial filtering technology (SFT) [11], photometric imaging [12], and microwave resonance technology (MRT) [13]. However, as these studies were conducted with different types of formulations and granulation units, the operational parameters of fluidized bed granulation have not received much recognition. Consequently, a comprehensive analysis of the granulation conditions and granule properties and compactability may be of great significance. The wet granulation in high-shear granulation and screw granulation has been studied extensively using DoEs and other nonlinear modeling techniques [14,15]. However, DoE has been rarely applied to better understand the effects of various variables on fluidized bed granulation characteristics [16]. Moreover, other empirical modeling techniques have been rarely applied.

In the QbD approach, modeling-based product development has replaced experiment-based product development [17,18]. Among the techniques used in statistical analysis, multivariate statistical technique allows a significant reduction in the number of experiments, and the effects of independent variables within the process are widely evaluated [19]. During the past decades, response surface methodology (RSM), partial least squares method (PLS) and artificial neural network (ANN) of multilayer perceptron (MLP) have been well established as modeling techniques and are the most relevant multivariate statistical techniques, providing an alternative technique where the mathematical relationship between the parameters and response of target is complex. To analyze different formulations, RSM, PLS and MLP are the most popular techniques and widely used in pharmaceutical research. The system can attain the best performance by using these techniques [3, 20–25].

RSM is a collection of mathematical and statistical techniques based on the fitting of a polynomial equation to the experimental data. This is based on the fitting of mathematical models such linear, square, polynomial, and others to the experimental results and further verifies the model using statistical techniques. The basic concept and application of RSM have been well reported [26]. However, the quadratic polynomial equation has poor estimates for highly nonlinear processes [27]. To overcome this limitation of the method, MLP has been used in the last few years in different processes because of its better estimates compared to RSM [28]. MLP provides an accurate prediction without a clear definition of the relationship between the input and output. Moreover, MLP has been investigated because of its easily understandable architecture and simple mathematical form, which is a convenient tool for modeling and optimization [29].

In our previous study, a multivariate statistical analysis model was successfully applied for modeling the granule yield in wet granulation [15]. Although the effects of process parameters on granule properties have been studied, a further detailed study is required to better understand the mechanisms involved during the granulation. Therefore, in this study, the critical factors for product variability affecting the characteristics of granules prepared by fluidized bed wet granulation were evaluated. In industrial pharmaceutical applications, it is common to use a mixture of different types of powder. MCC and lactose were selected because they represent commonly used excipients. The properties of these materials reflect the typical range of characteristics of pharmaceutical powders. Although a mixture of MCC and lactose was used in this study, the experimental design techniques and analysis methodologies are general and can be used for other powder systems.

In the first step, a Plackett-Burman design (PBD) combined with multivariate data analysis was performed to identify the high-risk variables affecting the properties of granules. In the second step, a Box-Behnken design (BBD) together with the RSM, PLS, and MLP was used to investigate the effects of high-risk variables. A combination of different process parameters with a predictable granulation behavior was determined using this procedure. The statistical model was experimentally tested to validate its robustness and accuracy within the design parameters.

## Materials and methods

### Materials

Sieved α-lactose monohydrate (Pharmatose^®^ 150M) supplied by DMV-Fronterra (Netherlands) and microcrystalline cellulose (MCC PH101) procured from Guangda Technological Development (China) were used as the fillers. A mixture of lactose and MCC in a ratio of 2:1 (wt/wt) was used as the granulation powder. Polyvinylpyrrolidone (PVP) (Plasdone K29/32) binder was kindly donated by Ashland Chemical Inc. (China). A two-component system water-PVP K29/32 (Plasdone) was used as the binder solution. Magnesium stearate (Sinopharm Chemical Reagent, China) was used as the lubricant during tableting.

### Characterization of primary materials

The particle size of lactose and MCC mixture was determined in triplicate using a Mastersizer 2000 laser diffraction particle size analyzer (Malvern Instruments Ltd., UK). The viscosity of the binder solution was measured experimentally using a rotary rheometer (MCR 101, Anton Paar, Austria) utilizing the cone and plate geometry (1°/50 mm) at a constant shear rate of 1 s^-1^ and a temperature of 25°C [30].

### Experimental design

#### PBD (1^st^ step) for the screening of high-risk factors

The PB DoE was performed and analyzed using the JMP software (version 10, SAS Inc., USA). In the preliminary experiments, the five factors investigated were: inlet air temperature (X_1_), atomization air pressure (X_2_), binder solution rate (X_3_), binder concentration (X_4_), and binder-to-powder ratio (X_5_). The independent and response variables selected in this design are listed in Table 1. Three center points were added to evaluate the potential curvature of the results. A total of fifteen experiments were conducted for five factors at three levels of each variable (Table 2). To minimize the effect of unexplained variability caused by the systematic errors, all the experiments were carried out at random.

**Table 1:** Processing conditions and values for the variables in the Plackett-Burman experimental design.

Factors (Independent variables)		Level in the experiments			Test formulation	
		Low (-1)	Medium (0)	High (+1)	T_1_	T_2_
Inlet air temperature	X_1_ (°C)	50	65	80	60	70
Atomization air pressure	X_2_ (bar)	0.6	1.0	1.4	1.0	1.0
Binder solution rate	X_3_ (g/min)	5.8	14.5	23.2	8.7	17.4
Binder concentration	X_4_ (%)	10	15	20	15	15
Binder-to-powder ratio	X_5_ (%)	8	11.5	15	10	14
Responses (dependent variables)						
Flowability (HR)	Y_1_					
Temperature (T)	Y_2_ (°C)					
Moisture content (MC)	Y_3_ (%)					
Aggregation index (AI)	Y_4_					
Compactability (Com)	Y_5_					

**Table 2:** The Plackett-Burman experimental design and response variables.

Run	Independent variables						Dependent variables				
	Mode	X_1_	X_2_	X_3_	X_4_	X_5_	Y_1_	Y_2_	Y_3_	Y_4_	Y_5_
PB-1	0	65	1.0	14.5	15	11.5	1.07	24.4	5.29	7.2	0.5293
PB-2	−−−+−	50	0.6	5.8	20	8.0	1.35	26.0	4.84	4.0	0.5018
PB-3	+−−−+	80	0.6	5.8	10	15.0	1.39	31.2	3.66	6.3	0.4484
PB-4	+−+++	80	0.6	23.2	20	15.0	1.03	29.3	4.31	7.9	0.4728
PB-5	−+++−	50	1.4	23.2	20	8.0	1.06	21.8	7.72	6.7	0.3588
PB-6	−−+−−	50	0.6	23.2	10	8.0	1.14	20.6	7.56	4.8	0.3553
PB-7	+++−−	80	1.4	23.2	10	8.0	1.06	28.1	4.9	4.6	0.4129
PB-8	−+−−+	50	1.4	5.8	10	15.0	1.18	21.9	4.72	3.5	0.5841
PB-9	0	65	1.0	14.5	15	11.5	1.05	25.2	7.58	7.1	0.5372
PB-10	0	65	1.0	14.5	15	11.5	1.03	26.4	4.72	7.1	0.5539
PB-11	−−+−+	50	0.6	23.2	10	15.0	1.01	23.0	6.86	9.0	0.5114
PB-12	−+−++	50	1.4	5.8	20	15.0	1.24	26.1	4.41	3.2	0.7602
PB-13	+−−+−	80	0.6	5.8	20	8.0	1.31	33.5	3.01	3.8	0.4832
PB-14	++−−−	80	1.4	5.8	10	8.0	1.34	29.7	3.43	2.8	0.5436
PB-15	+++++	80	1.4	23.2	20	15.0	1.01	27.9	5.38	8.3	0.5944

#### BBD (2^nd^ step) for the evaluation of high-risk factors

Based on the preliminary PBD screening experimental data, three variables (inlet temperature X_1_, binder solution rate X_3_, and binder-to-powder ratio X_5_) were selected as the high-risk factors. The BBD in RSM was performed for the evaluation study using the JMP software (version 10, SAS Inc., USA). The BBD in RSM is widely used for fitting a second-order model [31–33]. In this study, three-level and three-factor parameters with physical interpretation were defined by PB screening and used as the inputs for the DoE to model the agglomeration process. Fifteen runs, including three replicated center points, were used for each design in randomized order to study the effects of the three variables on the five response variables. The ranges of all the related parameters are shown in Table 3 (S1 and S2 Tables).

**Table 3:** The Box-Behnken design and results of the response variables.

Run	Independent variables			Dependent variables				
	X_1_	X_3_	X_5_	Y_1_	Y_2_	Y_3_	Y_4_	Y_5_
BB-1	50	14.5	8.0	1.3	23.6	6.28	4.8	0.390
BB-2	50	23.2	11.5	1.35	24.1	9.49	7.6	0.340
BB-3	80	5.8	11.5	1.34	32.5	3.27	4.2	0.533
BB-4	65	14.5	11.5	1.02	27.3	4.51	6.7	0.690
BB-5	80	14.5	8.0	1.05	29.8	3.75	5.9	0.482
BB-6	65	14.5	11.5	1.03	25.8	4.15	6.6	0.602
BB-7	65	23.2	8.0	1.15	27.1	6.05	6.0	0.416
BB-8	65	14.5	11.5	1.02	26.9	4.78	5.9	0.618
BB-9	65	5.8	8.0	1.49	29.4	3.82	3.8	0.485
BB-10	50	14.5	15.0	1.07	24.1	5.10	9.6	0.611
BB-11	65	5.8	15.0	1.26	32.3	3.28	4.4	0.599
BB-12	50	5.8	11.5	1.22	28.6	3.60	7.4	0.636
BB-13	80	23.2	11.5	1.01	30.2	4.49	7.6	0.420
BB-14	65	23.2	15.0	1.01	26.7	7.33	11.4	0.428
BB-15	80	14.5	15.0	1.02	30.0	3.32	8.1	0.522

### Preparation of granules

The granulation and drying were performed in a lab-scale batch fluidized bed granulator (FLZB 1.5, Chanse Ltd., China). The product chamber was made up of a conical cylinder with inner diameters of 16.0 cm and 33.0 cm at the bottom and top, respectively. The vertical height of the container was 54.0 cm. At the bottom of the container, an air distributor with a uniform distribution mesh made of a stainless steel plate was installed. At the top, a wind filter-bag was placed to prevent the flying of fine powder. A binder spray nozzle with 1.0 mm inner diameter was placed at 35 cm above the distributor. A binder solution was drawn and controlled using a peristaltic pump (Longer, China). The batch size was fixed at 300 g. A dehumidifier was coupled to control the humidity of the inlet air in the range 40–50%. The inlet fluidizing air velocity was adjusted manually in the range 40–90 m^3^/h to maintain the same level of fluidization state in each experiment about 25 cm above the bottom of the container, making it possible to have the same trajectory of particles during the granulation [34]. From the processing perspective, a constant inlet fluidizing air was inferior than a constant level of fluidization state. The main disadvantage of the constant inlet fluidizing air method was that it was prone to pneumatic delivery and entrainment at the early stage [35]. At the end of granulation, a de-fluidized phenomenon may occur due to large aggregations. Therefore, a constant inlet fluidizing air was not an efficient method to control the particle growth during the granulation in pharmaceutical manufacturing.

The particles were sieved using an 80-mesh (178 μm) sieve to prevent the cohesion between particles. After the sieving, the undersized fraction was reserved for the experiments. Before the experiments, MCC and lactose powders were mixed at a constant inlet fluidizing air (40 m^3^/h) for 5 min to achieve a uniform mixing effect. Experiments were carried out according to the design listed in Tables 2 and 3. Once the binder solution was consumed, the drying phase (inlet air temperature at 40°C) started and proceeded for 3 min to dry the surface moisture of the granules. After the granulation, the granules were collected and characterized.

### Property characterization

#### Flowability of granules

The flowability of granules was characterized by Hausner ratio (HR) following the literature [36]. The bulk granules were weighed and poured into a 100 mL graduated cylinder. The bulk and tapped densities were determined using a tapping machine (BT-100 Baite Corporation, China). As a surrogate for flowability, the HR was calculated from the bulk density (*ρ*_b_) and tapped density (*ρ*_t_) as shown in Eq 1.

$$\text{HR}=\frac{\rho_{\text{t}}}{\rho_{\text{b}}}$$ (1)

#### Temperature of granules

The temperature (T) of granules was recorded using a temperature probe inserted into the powder bed during the granulation. A thermocouple temperature probe was used to measure the temperature profile every 10 seconds during the granulation process. As the granulation liquid was gradually sprayed into the powders, the temperature of the material first decreased to an equilibrium value and then increased slightly. The granule temperature was considered to be the minimum value of the temperature curve.

#### Residual moisture content (MC) of granules

Approximately 2.0 g of each sample was used to determine the residual MC using a Sartorius MA35 instrument (Sartorius scientific Instrument, Germany) at a fixed temperature of 105°C. The thermal balance of the granule sample reached a constant weight at 5 min.

#### Granule size analysis

The size distribution of the dry granules in each batch was measured using a EML 200 digital plus T vibratory sieve shaker (Haver, Germany). The sieve analysis was performed using a series of sieves (90 μm, 125 μm, 180 μm, 250 μm, 355 μm, 425 μm, 710 μm, 850 μm, and 1180 μm). The granule samples (100 g) were placed on the shaker for 5 min at an amplitude of 1.5 mm. The amount of granules retained on each sieve was weighed to determine the size distribution of the granules. The mean particle size (d50) was interpolated by the cumulative particle size distribution, as shown in Eq 2. Furthermore, an aggregation index (AI) was proposed to evaluate the aggregation behavior as shown in Eq 3.

$$\text{d}50=\frac{\sum_{i=1}^{n}m_{i}\bar{X}}{\sum_{i=1}^{n}m_{i}}$$ (2)

$$\text{AI}=\frac{d50}{D50}$$ (3)

*m*_*i*_ is the mass retained on the sieve interval *X*_*i*_ to *X*_*i+1*_, $\bar{X}$ is the mean size of the size interval *i*. d50 is the mean granule size, and D50 is the mean size of the initial powder.

#### Compactability (Com) of granules

The compaction properties of the granules were tested by compressing the granules at different compression forces and determining the tablet hardness at each compression force using an automated tablet hardness tester (Sotax HT10, Switzerland). The granulated materials were compacted using a fully instrumented press (Korsch XP1, Germany) using an 8.5 mm, round, flat-faced tool with a compaction pressure of 2.0–7.0 kN while the tablet weight was 150 mg. In this study, the granules in the range 125–1180 μm were required for tableting as the target product has good flow properties, resulting in a high quality tableting behavior with a uniform tablet weight. The granule fraction of 125–1180 μm was compressed to evaluate the deformation potential of granules, avoiding the effects of fines and lumps. The upper punch, lower punch, and die-wall were lubricated with a 0.5% (w/v) magnesium stearate suspension in acetone prior to compaction. The thickness (*T*, mm), diameter (*D*, mm), and crushing force (*F*, N) of the tablets were assessed to calculate the diametral tensile strength (TS) as follows Eq (4):

$$\text{TS}=\frac{2F}{\pi DT}$$ (4)

The compaction profile was constructed using the TS value as the response variable, as a function of compression force in a dependent variable. A compactability parameter was determined as the slope of the initial linear part of the compaction profile and applied to the statistical analysis [37].

#### Shape and surface morphology

The surface morphology of the agglomerates was investigated by scanning electron microscopy (SEM) at an accelerating voltage of 20.0 kV (XL30 FEG ESEM, Philips, Netherlands). The Samples were fixed using a double-side adhesive carbon tape and sputter-coated with colloidal gold using a vacuum evaporator (EM ACE600, Leica, Germany). The representative granules obtained from 125–1180 μm size fraction were used for the morphological analysis in the two test formulations.

### Data statistical modeling

Three *in silico* techniques were used for the modeling and optimization of fluidized bed granulation: RSM, PLS and MLP.

#### RSM

A function of the independent variables was used to evaluate the properties of prepared granules, and the function, which was used for fitting the data and predicting the response, was a second-order polynomial model Eq (5) as follows [38]:

$$\text{Y}=\beta_{0}+\sum_{i=1}^{n}\beta_{i}X_{i}+\sum_{\begin{array}{l} i=1\\ i<j \end{array}}^{n-1}\sum_{j=2}^{n}\beta_{ij}X_{i}X_{j}+\sum_{i=1}^{n}\beta_{ii}X_{i}^{2}\text{+}\varepsilon$$ (5)

where *X*_1_, *X*_2_ and *X*_*n*_ are independent variables affecting the responses. Y, *β*_0_, *β*_*i*_, *β*_*ii*_, and *β*_*ij*_ are the regression coefficients for the intercept, linear, quadratic, and interaction terms, respectively. *n* represents the number of variables in the equation, and *ε* indicates the experimental error.

#### PLS

PLS modeling was carried out using SIMCA-P 11.0 (Umetrics, Umea, Sweden). PLS modeling relates two data matrices, of independent and dependent variables, to each other using a linear multivariate model [39]. Response contours were plotted for each of the dependent variables, along with the two independent variables that were selected based on the Variable Importance for the Projection (VIP) values. VIP values larger than 1 indicate important variables whereas values lower than 0.5 indicate unimportant ones [40].

#### MLP

**Fig 1:**
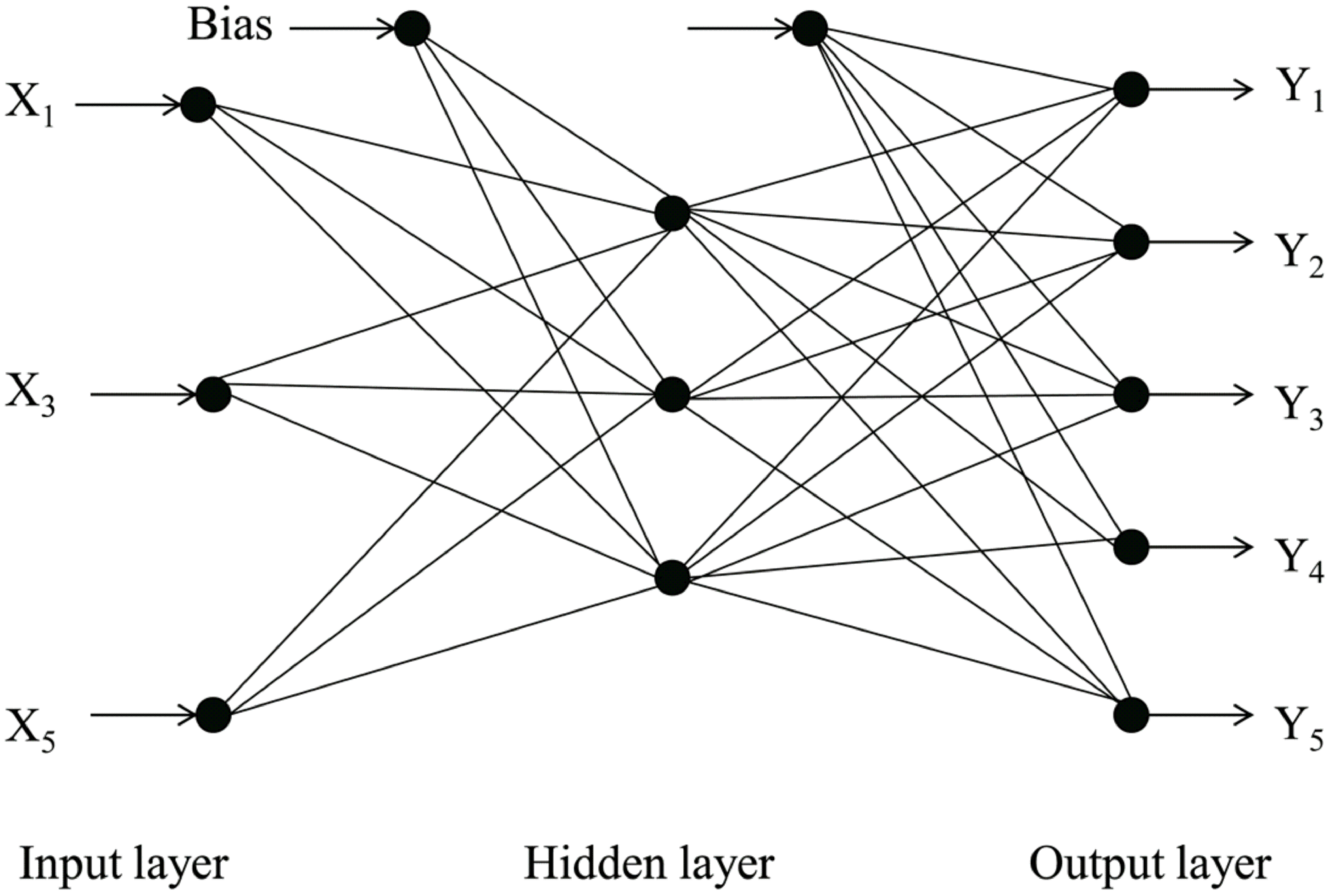
MLP neural network architecture used for modeling granule properties.

The ANN-MLP neural network was further considered as a good method for data set modeling to provide a nonlinear relationship between the input and output variables. The ANN-MLP architecture consisted of an input layer, an output layer, and a hidden layer. As shown in Fig 1, the independent input data were transmitted from the input layers to the output layer through the hidden layers in a single-hidden-layer feed-forward approach. The architecture of the MLP was multiple input-multiple output type. A commercial ANN software, Statistica 10 (StatSoft Inc., USA) was developed using a personal computer to design neural networks. The experimental data fed in the neural network were categorized into three sets: training (70%), testing (15%), and validation (15%). The input layer had three neurons because of three input variables X_1_, X_3_, and X_5_, whereas the output layer had five neurons. The connection between the neurons of the successive layers in an MLP network was established by connection weight (*ω*_*ij*_) and bias (*θ*_*j*_). The transition of data from the input variables to the hidden layer was obtained using the weights. Then, a neuron input (*I*_*j*_) was generated using the sum of these weight outputs (*X*_*i*_*ω*_*ij*_) and bias term (*θ*_*j*_) using Eq 6:

$${\text{I}}_{\text{j}}=\sum X_{i}\omega_{ij}+\theta_{j}$$ (6)

This neuron input was then passed through the output neuron along with a sigmoid transform function. The network was trained using the backpropagation algorithm, through 1000 epochs, with learning rate 0.3 and momentum 0.5.

## Results and discussion

### Material properties

The mean particle diameter (D50) of the lactose and MCC mixture was 52.31 μm, where those of D10 and D90 were 14.284 μm and 124.81 μm, respectively. The granulation liquid viscosities were 2.6 mPa.s, 17.2 mPa.s, and 25.9 mPa.s for 10%, 15%, and 20% concentrations of the PVP K29/32 solution, respectively.

### PBD screening study to identify the high-risk factors

PBD is a widely used screening design for the identification of high-risk factors that cause variability in product quality. The effects of the five factors considered in this study were statistically analyzed using PBD to identify the potential high-risk factors of the process in fluidized bed granulation.

**Fig 2:**
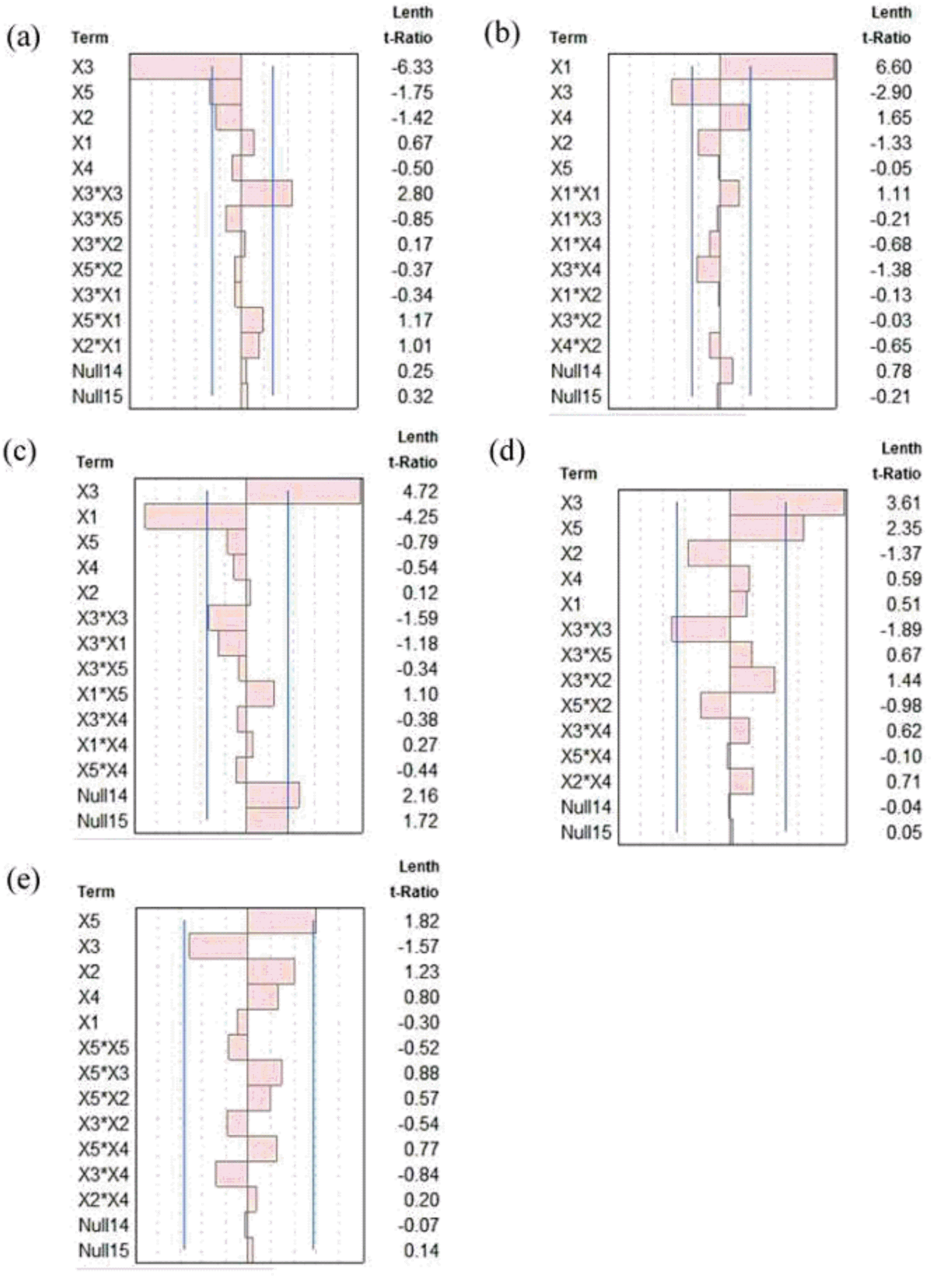
Standard Pareto chart showing the effects of various process factors on (a) flowability, (b) temperature, (c) moisture, (d) aggregation index, and (e) compactability.

Fig 2 shows the standardized Pareto charts of the main effects and their interactions. As shown in Fig 2a, the flowability (Y_1_) negatively correlated with the binder solution rate (X_3_) and binder-to-powder ratio (X_5_). As shown in Fig 2b, the temperature (Y_2_) positively correlated with the inlet temperature (X_1_) and negatively correlated with the binder solution rate (X_3_). The screening results of moisture are shown in Fig 2c, showing the opposite condition. For AI (Fig 2d), two variables, the binder solution rate (X_3_) and binder-to-powder ratio (X_5_), were significant factors with a positive effect. In the result of compactability (Y_5_), only one variable, binder-to-powder ratio (X_5_), was significant with a positive effect as shown in Fig 2e. Because of the nonsignificant effect of other factors on all the responses, the atomization air pressure (X_2_) and binder concentration (X_4_) were fixed at 1.0 bar and 15%, respectively, for the next stage of BBD.

### Mathematical modeling for granulation

The significant factors selected from PBD, namely, the inlet temperature (X_1_), binder solution rate (X_3_), and binder-to-powder ratio (X_5_) were considered for further analysis using BBD. The levels selected for the factors were set based on the previous PBD.

A multiple regression analysis was generated for each response variable by the polynomial relationships shown in Table 4 (S1 File). The parameter estimates are listed with the correlation coefficient (R^2^) and probability value (P). The sign and magnitude of the model parameter estimate are associated with the relative effects of each factor on the response. A larger magnitude of the model parameter indicated a more pronounced effect on the response, and the parameter estimate sign indicated that the independent and response were positive or negative correlation [37].

**Table 4:** Statistical analysis of variance (ANOVA) of the response (Y_1_-Y_5_) results.

Factors	HR (Y_1_)		T (Y_2_)		MC (Y_3_)		AI (Y_4_)		Com (Y_5_)	
	Coefficient	P-Value	Coefficient	P-Value	Coefficient	P-Value	Coefficient	P-Value	Coefficient	P-Value
Intercept	1.02	<0.0001	26.67	<0.0001	4.48	<0.0001	6.40	0.0001	0.64	<0.0001
X_1_	-0.07	0.0375	2.76	<0.0001	-1.21	0.0008	-0.45	0.2754	-0.01	0.8824
X_3_	-0.10	0.0079	-1.84	0.0006	1.67	0.0002	1.60	0.0073	-0.08	0.0039
X_5_	-0.08	0.0191	0.40	0.1572	-0.11	0.5420	1.63	0.0069	0.05	0.0297
X_1_X_3_	-0.12	0.0170	0.55	0.1668	-1.17	0.0042	0.80	0.1844	0.05	0.1002
X_1_X_5_	0.05	0.1868	-0.08	0.8342	0.19	0.4615	-0.65	0.2665	-0.05	0.1031
X_3_X_5_	0.02	0.5221	-0.83	0.0597	0.46	0.1108	1.20	0.0691	-0.03	0.3128
X_1_^2^	0.04	0.2472	0.09	0.8060	0.11	0.6651	0.50	0.3979	-0.07	0.0355
X_3_^2^	0.16	0.0051	2.09	0.0020	0.62	0.0524	-0.20	0.7268	-0.09	0.0144
X_5_^2^	0.04	0.2713	0.12	0.7551	0.02	0.9381	0.20	0.7268	-0.07	0.0351
R Square	0.94		0.98		0.97		0.91		0.93	
RMSE	0.06		0.68		0.47		1.04		0.05	

**Fig 3:**
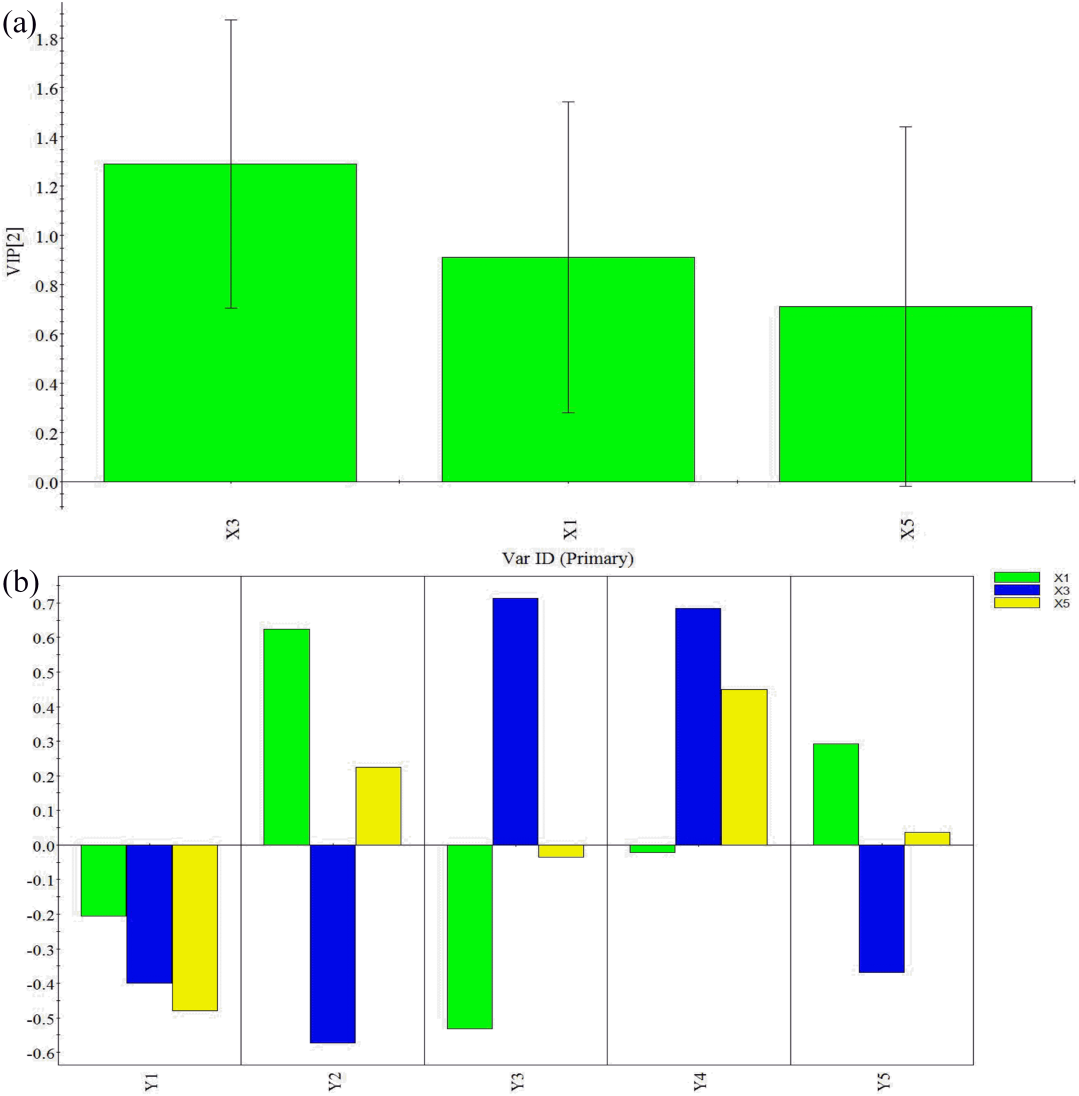
(a) VIP and (b) coefficients for the PLS model.

As mentioned previously, in this study, two other models MLP and PLS were also used. In this study, the MLP network was proposed based on the sigmoid activation function with three neurons in the hidden layer for the five responses. The learning rates were set to 0.1, 0.2, and 0.3 and the simulation momenta were set to 0.3 and 0.5 [24]. To investigate the results of the MLP simulation, three figures of merit were calculated, namely training, testing, and validation R^2^ values. This was done for each different learning rate and momentum. The R^2^ values for training, testing, and validation all increased with the increasing learning rate and momentum within the chosen ranges. When the learning rate was 0.3 and the momentum was 0.5, the R^2^ values for training, test and validation were 0.9, 1.0 and 0.6, respectively, which meant that the simulation result was more reliable. The error for the training set was 2.09, for the test set was 3.44, and for the validation set was 6.46 (S2 File). The R2Y (cum) value, i.e., the correlation between the measured and predicted values for the response under study, and the Q2 (cum) value, i.e., the correlation between the measured and cross-validated predicted response, can be used to evaluate the veracity and quality of a PLS model. Higher R2Y (cum) and Q2 (cum) values imply a better response, which can be fitted and predicted as a function of the descriptor variables [41]. With the PLS model, the values of R2Y (cum) and Q2 (cum) were 0.58 and 0.31, respectively. R2Y (cum) is the fraction of the variance of the Y variables that is explained by the extracted components. In general, R2Y (cum) greater than 0.5 indicates a good fit. The PLS model explained 58% of the variation of independent variables. Q2 (cum) is the fraction of the total variation of the Y variables that can be predicted by the components. Q2 (cum) is also described as the cross-validated variance statistic, and is significant when larger than a critical value (Q2 limit = 0.097) that corresponds to p < 0.05 [42]. Thus, the established PLS model for the variability in the fluidized bed granulation process was acceptable (S3 File). The VIP values and coefficients for the PLS model are shown in Fig 3. Contours were plotted for each dependent variable, along with the two independent variables chosen based on their VIP values. It can be seen that binder solution rate and inlet air temperature have more pronounced influences on granule properties and that binder-to-powder ratio was the least important factor for granule properties. Therefore, the counter plot was based on binder solution rate and inlet air temperature.

#### Effects of various factors on granule flowability

HR provides an indication of the flow behavior; it also provides a measurement of the packing behavior of granules. The flowability of powder materials was classified from excellent (1.00–1.11), good (1.12–1.18), fair (1.19–1.25), to passable (1.26–1.34) [43].

**Fig 4:**
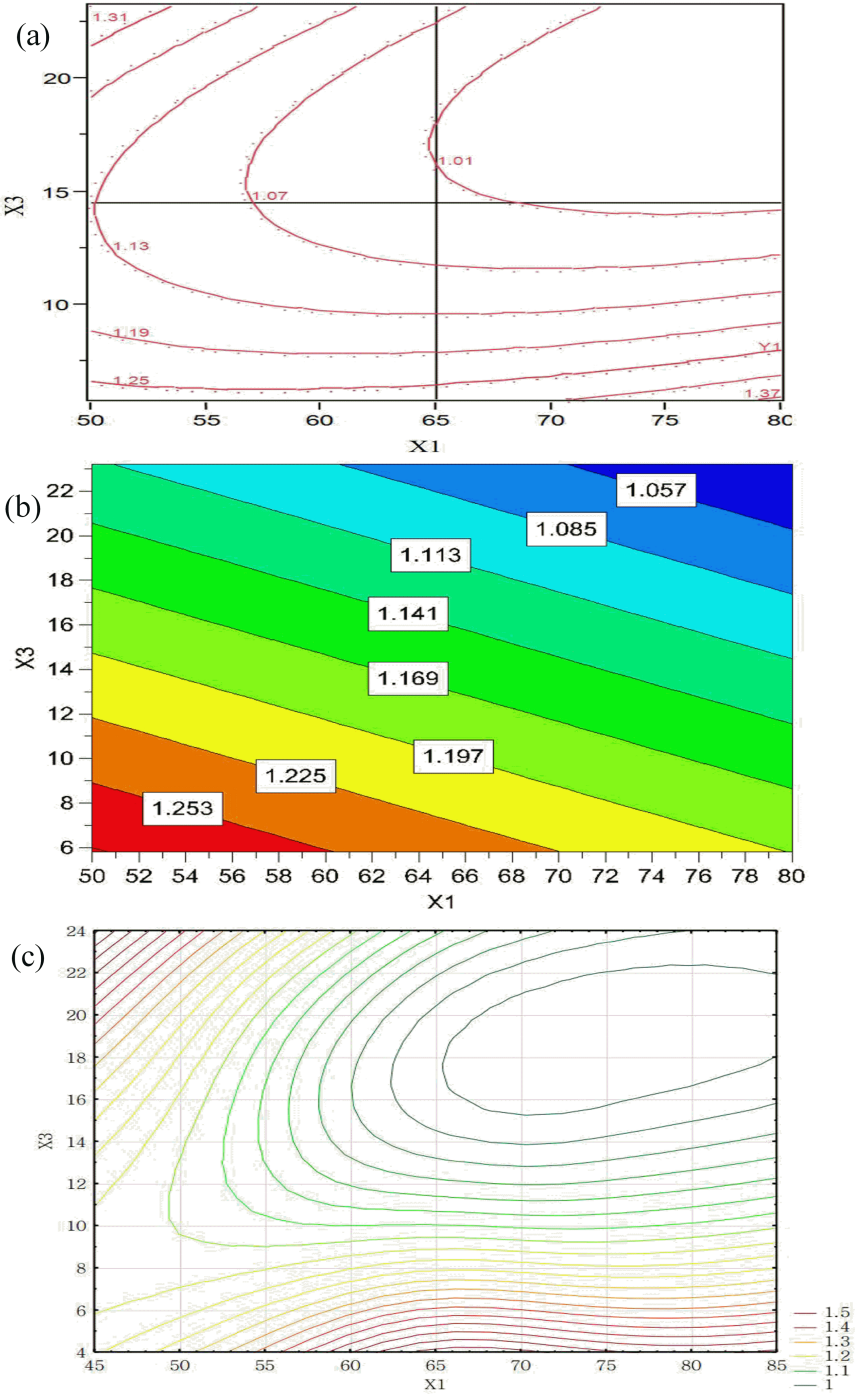
Contour plots showing the effects of X_1_ and X_3_ on HR obtained by using: (a) RSM, (b) PLS, and (c) MLP (X_5_ = 11.5).

The results indicate that a rather wide HR in the experimental setting range was obtained in the range 1.01–1.49 (Table 3). The statistical analysis results in RSM show that three factors significantly affected the granule flowability (Table 4). By increasing X_1_, X_3_ and X_5_, the HR of granules was decreased. The interaction effects of X_1_ and X_3_ were favorable for reducing the HR value. The contour plots generated using the RSM, PLS and MLP are shown in Fig 4. These plots were useful in studying the effects of two factors on the response at one time. Moreover, the interaction effects of the factors in RSM and MLP were also included. The results of the three models indicate that HR is mostly affected by the X_3_ (Fig 4a and 4c). The elliptical of the contour plot showed the significance of the interaction parameters on the response. MLP predicted a more complex effect of the investigated factors with a minimum value of HR (Fig 4c). In the upper right corner of the Fig 4a and 4c, the minimum HR values were observed after applying constraints to X_1_ (>65°C) and X_3_ (<15 g/ml). Better flow properties of the granules could be related to smoother granule surfaces, corresponding to the higher degrees of granule circularity.

#### Effect of various factors on granule temperature

Relatively low values of granule temperature (T), ranging from 23°C to 32°C, are shown in Table 3. Granule T affects the ability of the binder liquid to evaporate and the aggregation of the particles. Granule T is very important, not only impacting granule nucleation and growth, but also causing drug instability. If the temperature is low, there can be poor evaporation efficiency, inducing larger lumps and causing further overwetting and de-fluidization. There are few reports about the temperature of granules during granulation. From the point of view of processing, it is reasonable to control the temperature of granules at around 30°C during the granulation process [44].

**Fig 5:**
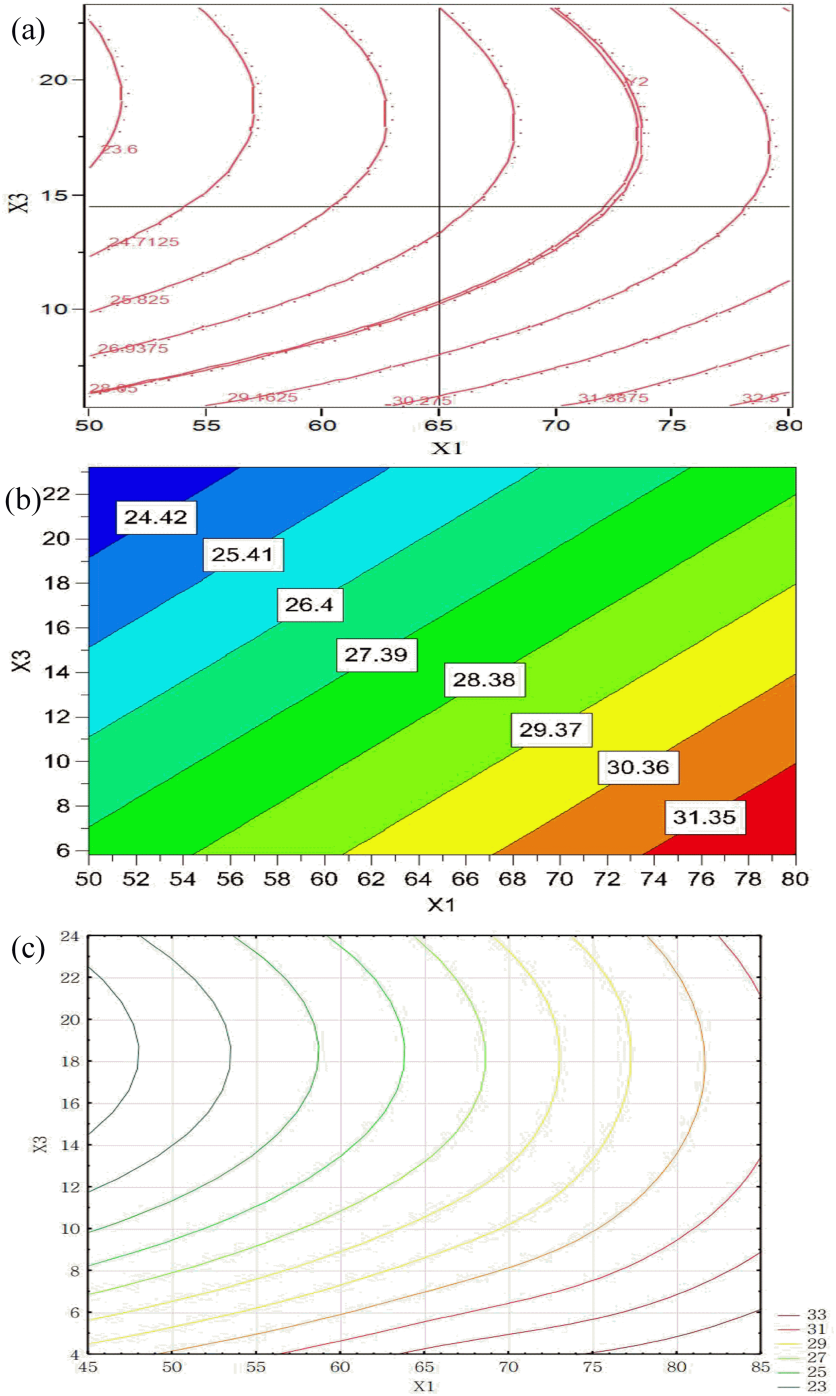
Contour plots showing the effects of X_1_ and X_3_ on granule temperature obtained by using: (a) RSM, (b) PLS, and (c) MLP (X_5_ = 11.5).

From the regression equation in Table 4, it is evident that X_3_ had direct negative and quadratic effects on the granule T while X_1_ was able to increase granule T. Fig 5 shows contour plots for the combination of parameters of X_1_ and X_3_ at the medium value of X_5_ (11.5%). Similar results were obtained in all three models. Fig 5 shows that the inlet air temperature remarkably affected the temperature of granules with a synergistic effect, whereas the rate of granulation liquid was less important with a negative effect. Granule temperature decreased by increasing the rate of granulation liquid and decreasing the inlet air temperature. This is because a high amount of liquid was input, and heat was absorbed due to liquid evaporation. On the other hand, it was expected that preheated air was the source of energy. At a higher inlet air temperature, heat was more rapidly transferred to the granule material, resulting in a high granule T.

#### Effects of various factors on granule residual MC

The values of MC ranging from 3.0% to 9.5% are shown in Table 3. This indicates the differences in the moisture retention capacity of different batches of granules. Relatively low MC values were obtained for all granulates, except for granulate BB-2 (Table 3). Heated air with low relative humidity encounters the surface of the fluidized wet particles that transfer heat into the solid by conduction. Then the moisture on the surface or migrating from the inner core is transported away by air convection. A multi-faceted effect of MC could be applied to the material properties. Electrostatic forces are weakened by increasing moisture content because of the conductive properties of water, while friction and interlocking caused by surface roughness, are also decreased by moisture through the lubricant effect [45]. However, increasing the moisture content can strengthen the liquid bridges formed between particles by increasing the thickness of the liquid layer. Furthermore, moderate moisture contributes to the compactibility of tablet without capping, lamination or breaking up. However, there is no standard moisture content that has been universally accepted for pharmaceutical processes. In fact, the amount of material MC depends on the properties of materials and the preparation process.

**Fig 6:**
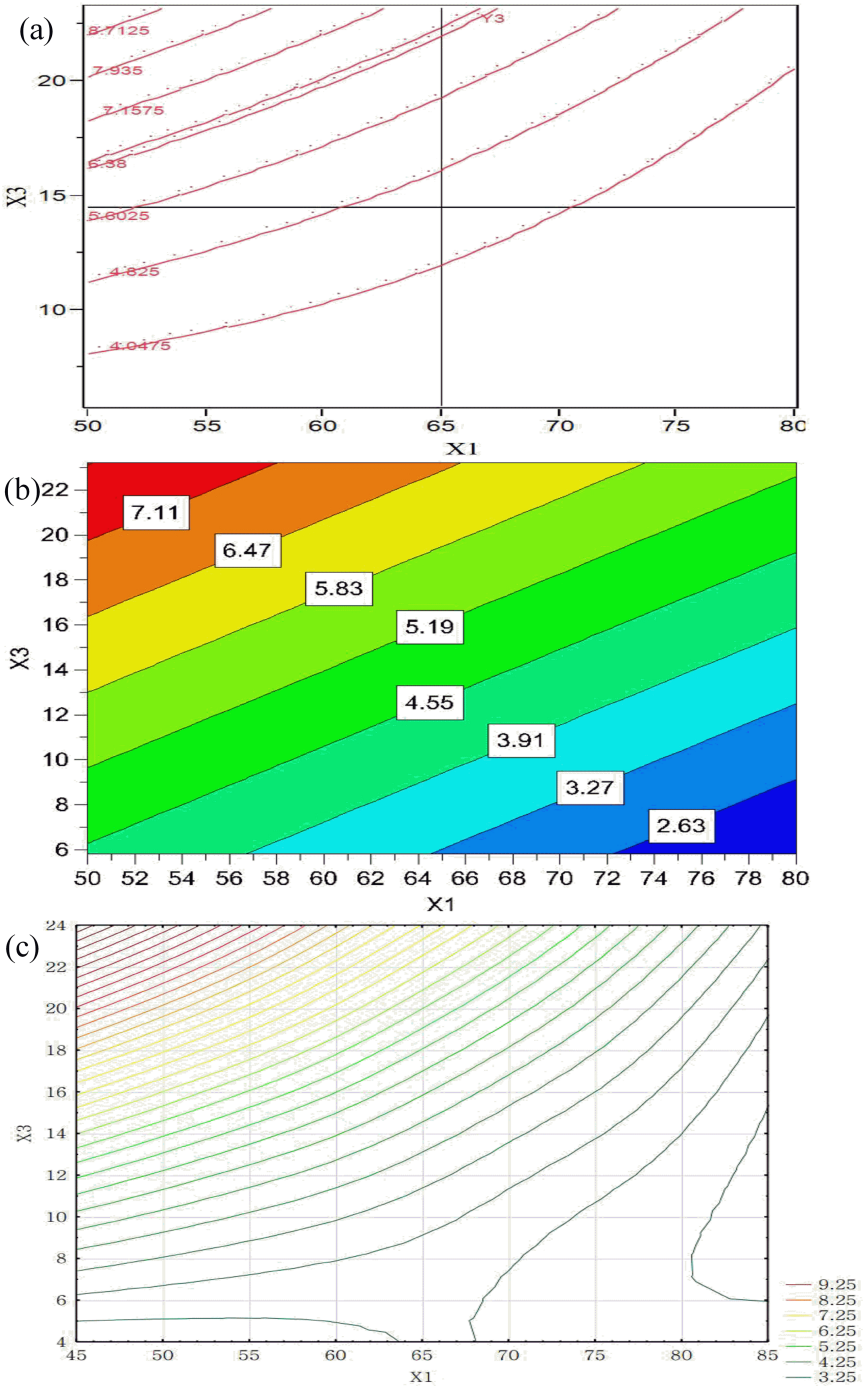
Contour plots showing the effects of X_1_ and X_3_ on granule residual MC obtained by using: (a) RSM, (b) PLS, and (c) MLP (X_5_ = 11.5).

According to the results of the statistical analysis obtained by RSM, X1 and X3 showed the most significant effects on MC (Table 4). MC was also significantly influenced by the interactions between X1 and X5. The plots showed the effects of X_1_ and X_3_ on MC (Fig 6). It can be concluded that an increase in X_3_ and a decrease in X_1_ resulted in a higher MC of granules in both the models (RSM, PLS and MLP). An opposite effect of these factors was observed on granule temperature. This was expected. An increase in X_1_ facilitated the evaporation of the liquid as well as promoted heat transfer from air to the granules. The results are consistent with those of other groups: The inlet air temperature is directly proportional to the MC of granules [46].

#### Effects of various factors on AI of granules

The AI results show that a large granule size ranging from 4 to 11 was obtained from fluidized bed granulation with appropriate operating parameters (Table 3). Parameter estimation in RSM indicates that the most effective operating parameters to control powder aggregation were X_3_ and X_5_ in which a wide range of granule mean size form ~200 μm to 600 μm was obtained.

**Fig 7:**
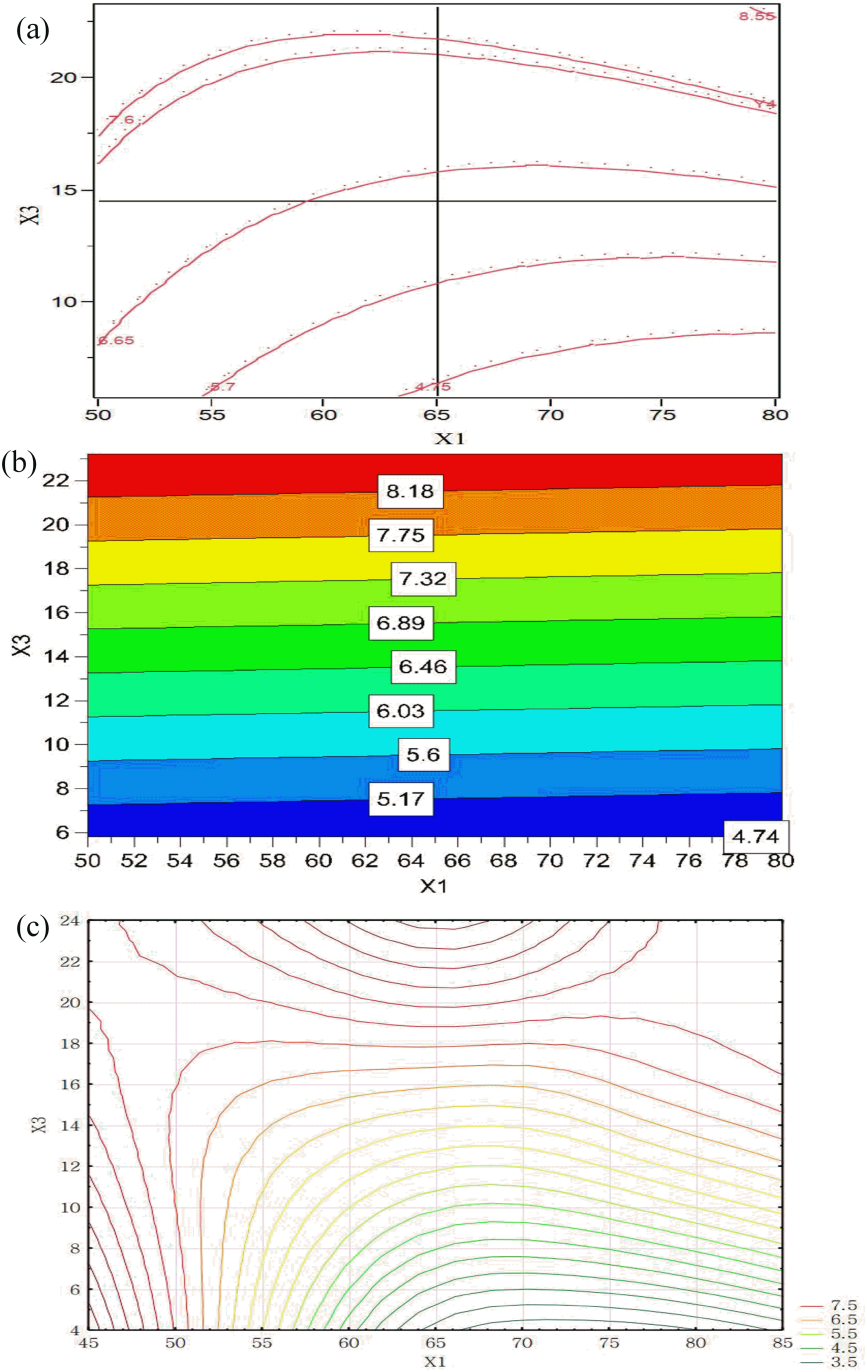
Contour plots showing the effects of X_1_ and X_3_ on AI obtained by using: (a) RSM, (b) PLS and (c) MLP (X_5_ = 11.5).

Fig 7 shows the contour plots of the response variables. These plots showed a variation in the response with respect to each factor. A high X_3_ would result in a large size of granule samples (Fig 7). The increase in the size of granules can be attributed to the increase in the availability of granulation liquid with a higher X_3_; more droplets were distributed in the powder surface to accelerate the aggregation between particles. A higher X_3_ led to a larger number of droplets sprayed. Therefore, more binder dispersed on the particle surface, and larger moist zones were available for bridge formation, thus forming agglomerates within a short period of time [47]. The size increase and growth showed a different sensitivity to X_1_ and X_3_ during the agglomeration in the RSM and MLP models (Fig 7a and 7c). The contour plot obtained by PLS model (Fig 7b) shows that X_1_ has only a small effect on powder aggregation behavior.

#### Effects of various factors on granule compactability

The TS increased with the increase in compaction pressure. In a relatively low compaction pressure (2.0–7.0 kN), a linear relationship was obtained. The statistical analysis results show that X_3_ and X_5_ significantly affected the granule compactability (Table 4). However, the results obtained using PBD were interesting: X_5_ was the only critical factor (Fig 4e). This is probably because the compactability of granules is a complex process involving elastic–plastic deformation and is affected by many factors such as particle size, granule solid fraction, and MC [48,49].

**Fig 8:**
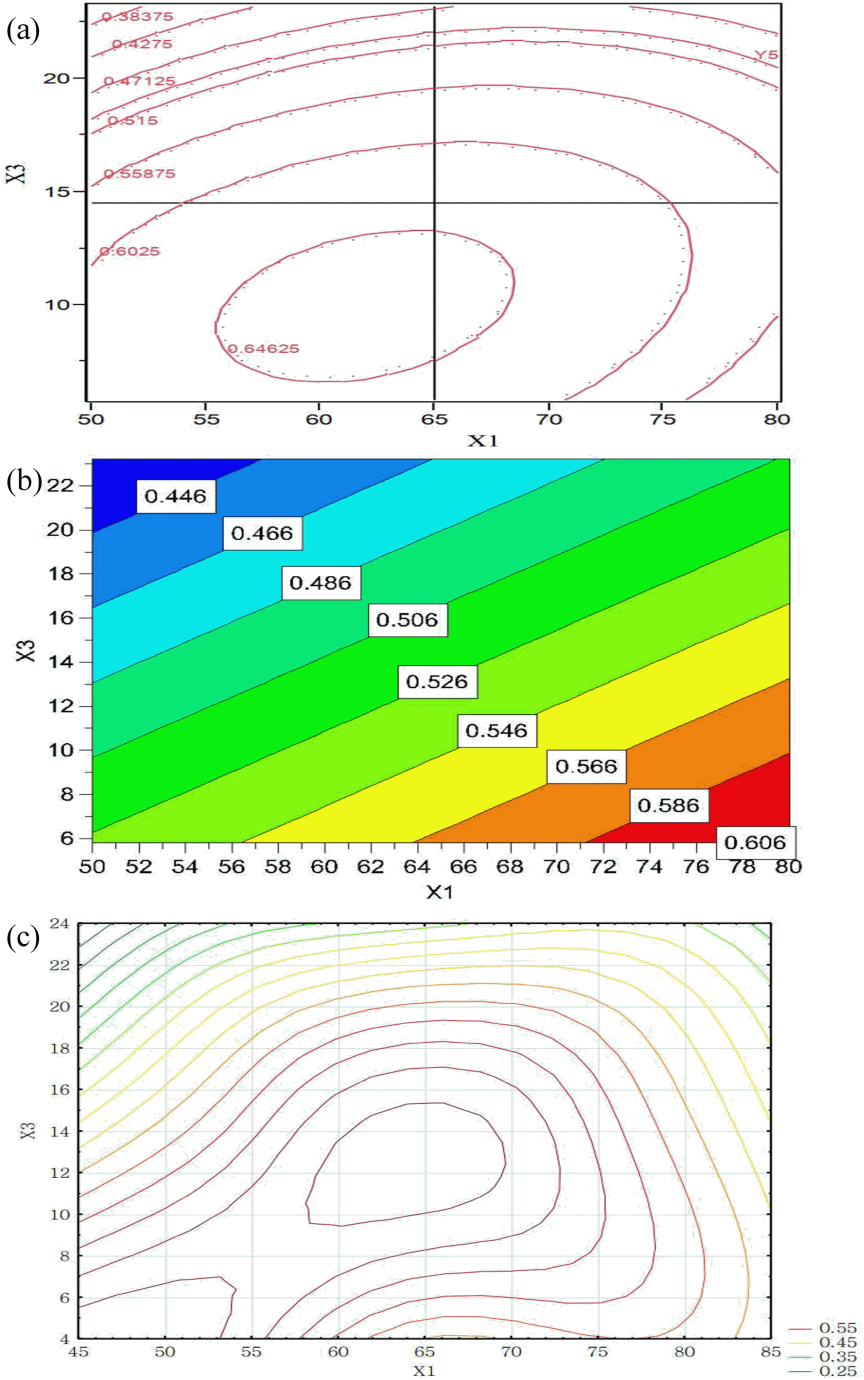
Contour plots showing the effects of X_1_ and X_3_ on granule compactability obtained by using: (a) RSM, (b) PLS, and (c) MLP (X_5_ = 11.5).

The contour plots generated using the RSM, and MLP models show that the compactability increased from 0.4–0.6, but slowly decreased when the duration was extended with a high X_3_ (Fig 8). The plot from the PLS model shows that higher X_1_ leads to higher granule compactability (Fig 8b), while RSM and MLP analyses predict higher compactability at relative lower X_1_ (Fig 8a and 8c). X_3_ significantly affected the compactability in both the models. This may be responsible for the effect of aggregation, indicating that a higher X_3_ produced softer tablets with a low hardness. The amount of binder and concentration in granulation would affect the granule aggregation. Because tablets are mainly bonded together with van der Waals attraction, an increase in the granule size would reduce the surface area of the contact, directly affecting the crushing strength of the tablets [50]. A higher X_3_ led to a higher MC of granules with a worse mechanical strength. This is probably because higher moisture levels remarkably reduced the mechanical strength of the tablets, and the MC strongly affected the elasticity and plasticity of granules during the compaction [51].

Evaluations of the prediction performance for the different models (RSM, PLS, and MLP) were based on testing for correlations between measured and predicted values. Table 5 shows the results for predicted and observed values using the different models (S3 Table). The MLP- and PLS-based models exhibited similar sensitivity in observed vs. predicted correlations. It is clear that using RSM-based evaluation results in a better correlation with the data than PLS or ANN. This could be due to the fact that PLS and ANN functions may not suitable for modeling a complex surface since they cannot detect slight changes in parameters and considerably under/overestimate the values in those regions where the response function changes its sense [52]. In this study, it has been shown that the nonlinear approach based on RSM is better at generalization and prediction than the multiple linear technique PLS. However, this method has some disadvantages. For example, the quadratic polynomial function gives poor estimates for highly nonlinear processes and overlapping mean responses do not account for the uncertainty in the DoE parameters [27,53]. The use of MLP also has some drawbacks in that specific analytical formulae could not be obtained, the accuracy of the results are uncertain, and a highly predictive model cannot be interpreted with respect to its physical meaning. Therefore, there is no ideal model and the selection of appropriate model should be based on the experimental data and consideration of how the model will be applied.

**Table 5:** Linear regression on the predicted vs. measured data.

	RSM		PLS		MLP	
	Equation	R^2^	Equation	R^2^	Equation	R^2^
Y_1_	y = 0.9391x+0.0704	0.9391	y = x-2E-14	0.4594	y = 0.9398x+0.1054	0.7399
Y_2_	y = 0.9793x+0.5774	0.9793	y = x+3E-05	0.7992	y = 0.9003x+3.332	0.6737
Y_3_	y = 0.9743x+0.1253	0.9743	y = x+3E-06	0.7919	y = 0.8521x+0.6943	0.8049
Y_4_	y = 0.9089x+0.6016	0.9089	y = x-3E-06	0.7217	y = 0.5380x+3.2099	0.5485
Y_5_	y = 0.9332x+0.0346	0.9332	y = x+3E-07	0.4620	y = 0.2664x+0.3429	0.3703

### Model validation

Two test formulations obtained under the experimental conditions shown in Table 1 were prepared and analyzed to assess the prediction ability and accuracy of the developed models using RSM, PLS and MLP. Table 6 lists the experimental responses and predicted values using RSM, PLS and MLP for test formulations T_1_ and T_2_. The values obtained from the RSM, PLS and MLP were similar to the experimental data of the percentage prediction error (PE). High prediction ability among these techniques indicates that the granulation could be controlled using simple variables to achieve the desired granule properties.

**Table 6:** Experimental values (EV) and predicted values (PV) of response variables obtained for test formulations.

			HR	T	MC	AI	Com
T_1_		EV	1.12	27.63	4.15	5.5	0.705
	RSM	PV	1.22	27.74	3.98	5.2	0.608
		PE	-8.93	-0.40	4.10	5.45	13.76
	PLS	PV	1.27	28.18	4.22	4.9	0.537
		PE	-13.39	-1.99	-1.69	10.91	23.83
	MLP	PV	1.31	29.90	4.22	5.6	0.488
		PE	-16.96	-8.22	-1.69	-1.82	30.78
T_2_		EV	1.01	27.41	5.26	8.4	0.493
	RSM	PV	0.96	27.41	4.67	8.3	0.579
		PE	4.95	0	11.22	1.19	-17.44
	PLS	PV	1.04	28.55	4.96	8.2	0.518
		PE	-2.97	-4.16	5.70	2.38	-5.07
	MLP	PV	1.00	29.30	4.15	8.1	0.513
		PE	0.99	-6.90	21.10	3.57	-4.06

However, in most cases, the PLS and MLP showed a significant deviation in prediction according to the values of PE, indicating a better prediction ability of RSM than PLS and MLP. The difference in prediction ability is due to the fact that the calculation criteria are different in different models [54].

### Shape and surface morphology

**Fig 9:**
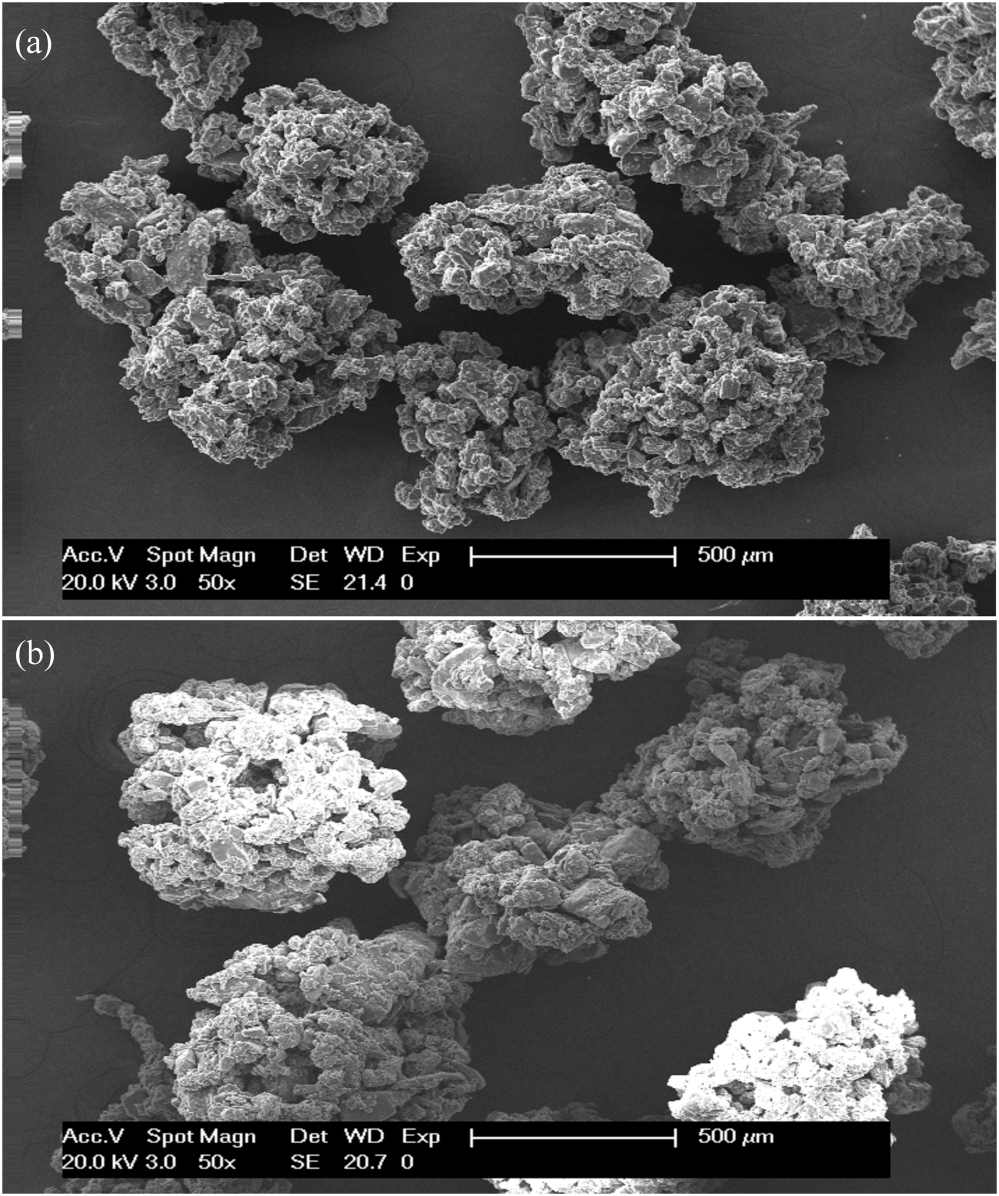
SEM micrographs of: (a) granules obtained by T_1_ and (b) granules obtained by T_2_.

The investigated surface of the validated granules (125–1180 μm) using SEM showed a wrinkled surface with a narrow size distribution (Fig 9). Moreover, the formed granules were almost spherical and porous shaped, indicating that distribution nucleation was the dominant agglomeration mechanism at the investigated parameters [55].

## Conclusions

The experimental data obtained in this study show that the high-risk factors in fluidized bed granulation were successfully screened and identified using PBD and BBD. Furthermore, multivariate modeling was an efficient tool in the mechanistic understanding of the influence of the investigated variables on the quality attribute of the prepared granules. A study on fluidized bed granulation was successfully conducted using a two-step approach.

This study demonstrates the usefulness of the QbD methodology to fundamentally understand the critical factors of fluidized bed granulation. The results of the statistical analysis show that the RSM model had a better ability to fit the quality attribute of granules than the PLS and ANN models. This study confirmed that RSM, PLS, and MLP are valid alternative approaches to modeling complex systems. These models were successfully used to develop fluidized bed granulation in the early formulation development stage and provide a useful tool to predict the quality attributes according to different process parameters using a minimal amount of experiments.

## Supporting information

S1 Table Relevant data for Hausner ratio (HR) in Box-Behnken design and model validation experiments. Arithemtic mean values and standard errors (SE) for HR were calculated.
(XLSX)

S2 Table Data used in compactability of granules. Compactability profile was constructed using the tensile strength (TS) value as a function of compression force.
(XLSX)

S3 Table Data for linear regression analysis. Linear regression on the predicted vs. measured data.
(XLSX)

S1 File Data statistical modeling for response surface methodology (RSM). Statistical analysis of variance (ANOVA) of Y1-Y5 responses.
(DOCX)

S2 File Data statistical modeling for artificial neural network (ANN) of multilayer perceptron (MLP). Results of PLS analysis of Y1-Y5 responses.
(DOCX)

S3 File Data statistical modeling for partial least squares method (PLS). Overview of the PLS model.
(DOCX)
