# Supplementary material for: A two-step approach for fluidized bed granulation in pharmaceutical processing: Assessing different models for design and control
Source: PLoS One. 2017 Jun 29;12(6):e0180209. doi: 10.1371/journal.pone.0180209 (PMC5491152; doi:10.1371/journal.pone.0180209)

**Data statistical modeling for response surface methodology (RSM).** Statistical analysis of variance (ANOVA) of Y1-Y5 responses. The Box-Beheken design in RSM was performed for the evaluation study using the JMP software (version 10, SAS Inc., USA)


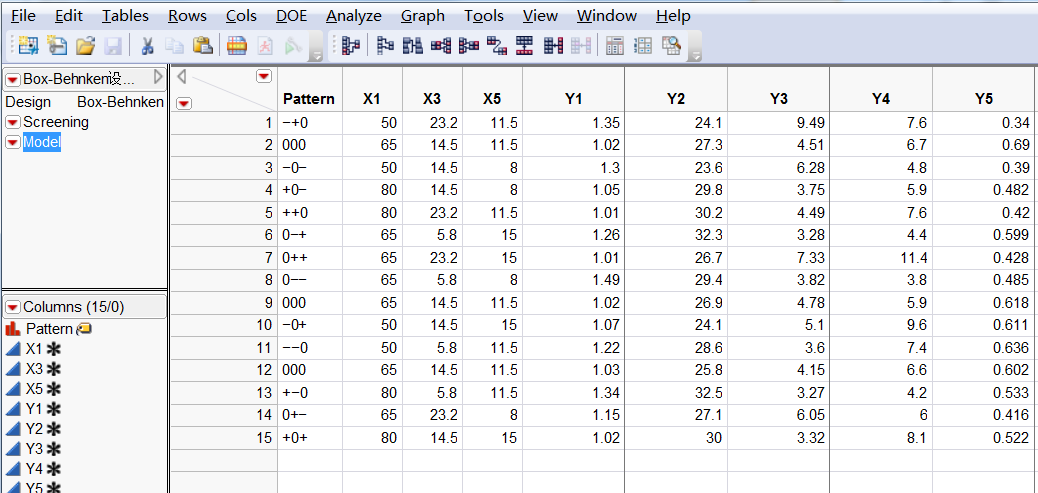


Statistical analysis of ANOVA of the Y1 response


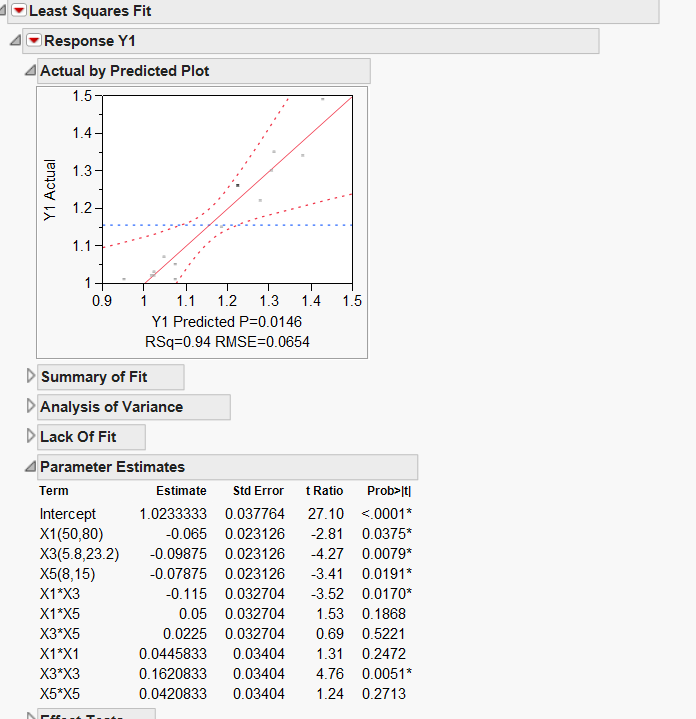


Statistical analysis of ANOVA of the Y2 response


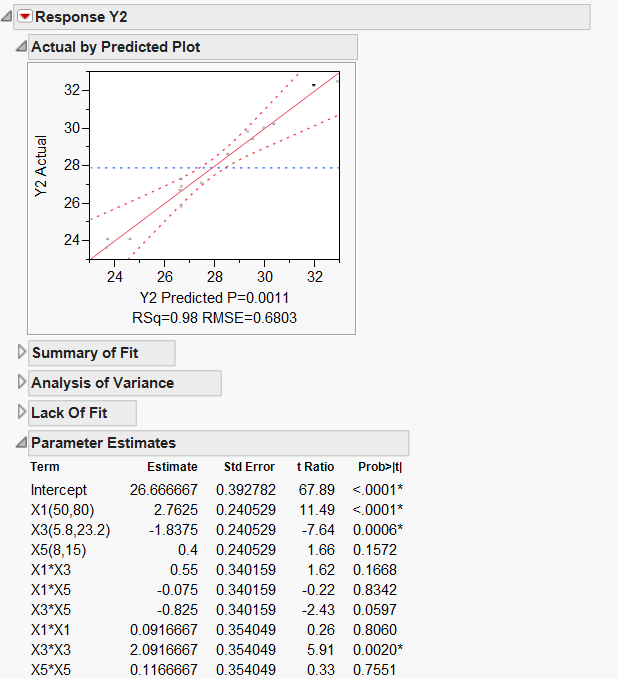


Statistical analysis of ANOVA of the Y3 response


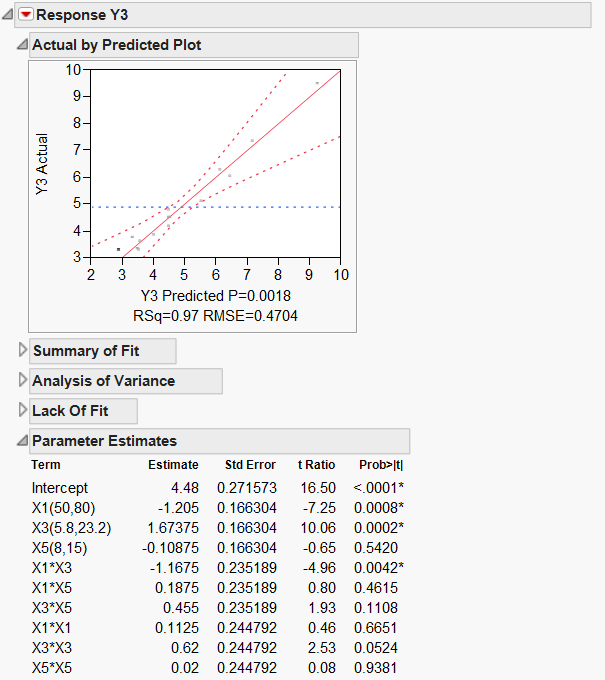


Statistical analysis of ANOVA of the Y4 response


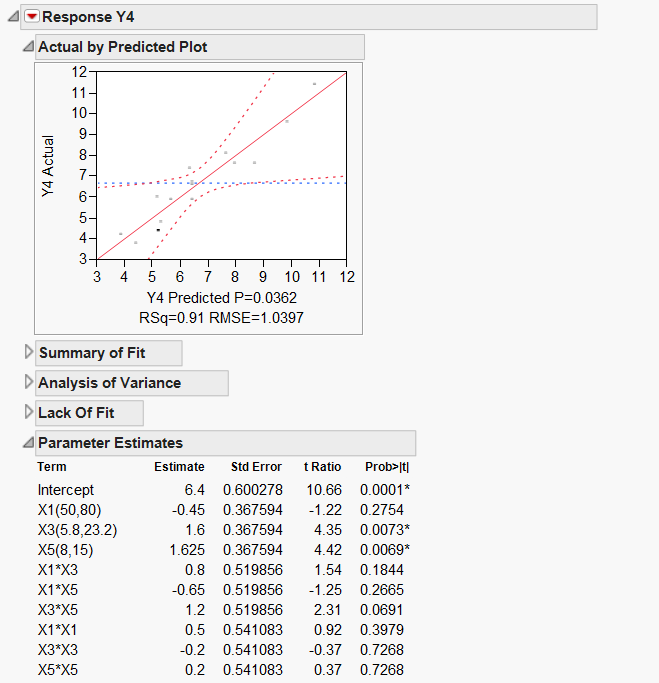


Statistical analysis of ANOVA of the Y5 response


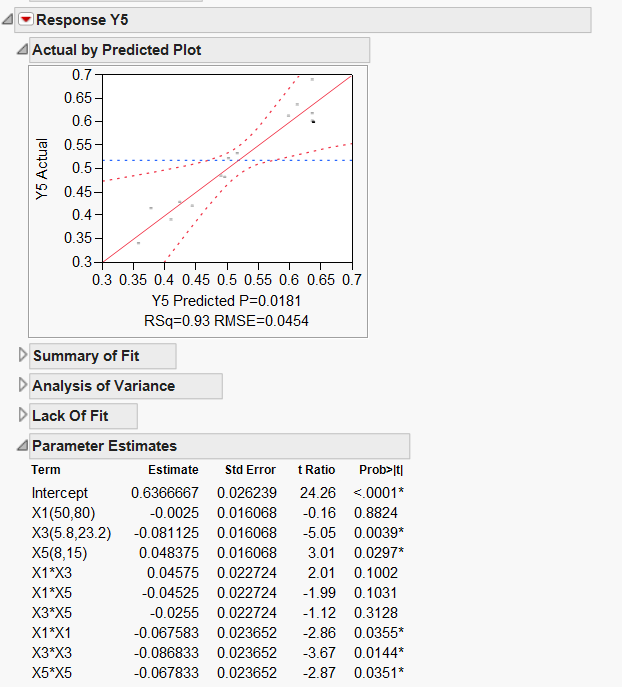

Supplement: S1 File — Statistical analysis of variance (ANOVA) of Y1-Y5 responses. (DOCX) [file pone.0180209.s004.docx]
