# Supplementary material for: A two-step approach for fluidized bed granulation in pharmaceutical processing: Assessing different models for design and control
Source: PLoS One. 2017 Jun 29;12(6):e0180209. doi: 10.1371/journal.pone.0180209 (PMC5491152; doi:10.1371/journal.pone.0180209)

**Data statistical modeling for artificial neural network (ANN) of multilayer perceptron (MLP).** Results of PLS analysis of Y1-Y5 responses (docx)


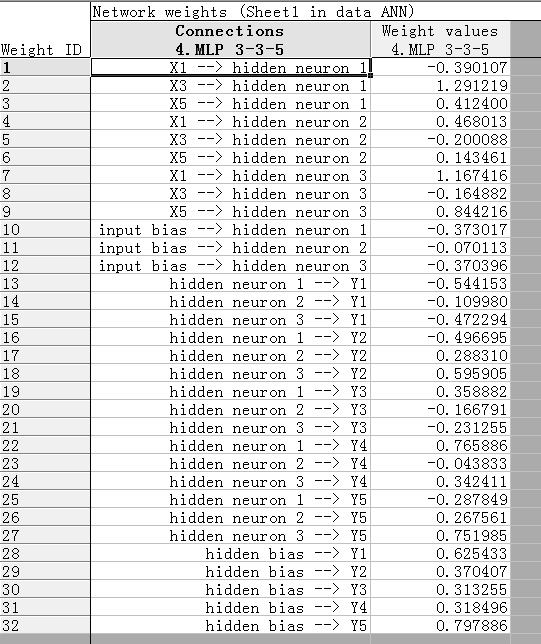


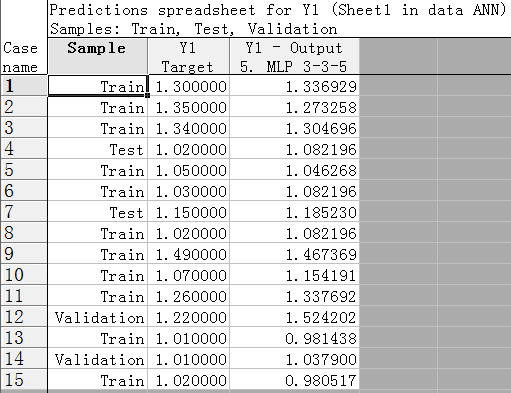


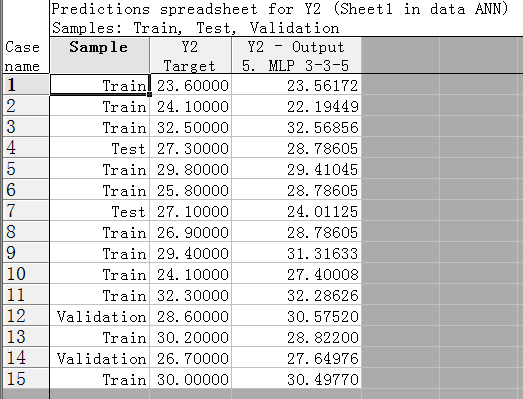


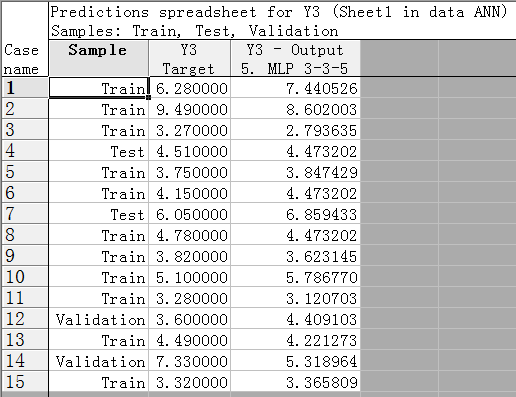


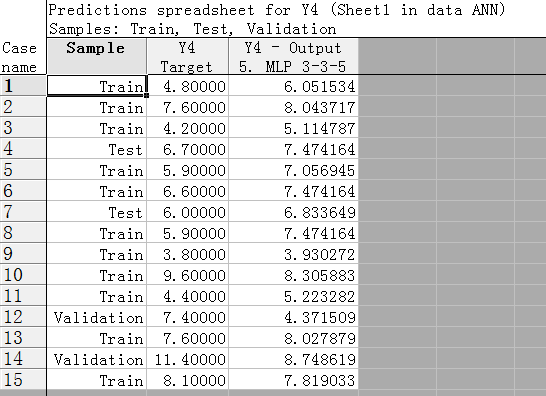


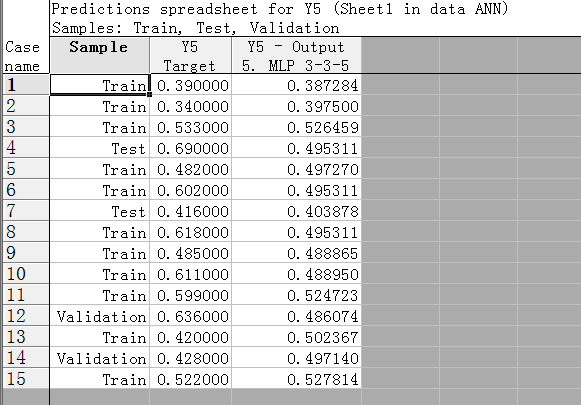

Supplement: S2 File — Results of PLS analysis of Y1-Y5 responses. (DOCX) [file pone.0180209.s005.docx]
