# Supplementary material for: A two-step approach for fluidized bed granulation in pharmaceutical processing: Assessing different models for design and control
Source: PLoS One. 2017 Jun 29;12(6):e0180209. doi: 10.1371/journal.pone.0180209 (PMC5491152; doi:10.1371/journal.pone.0180209)
Supplement: S3 File — Overview of the PLS model. (DOCX) [file pone.0180209.s006.docx]

**Data statistical modeling for partial least squares method (PLS).** Overview of the PLS model (docx).


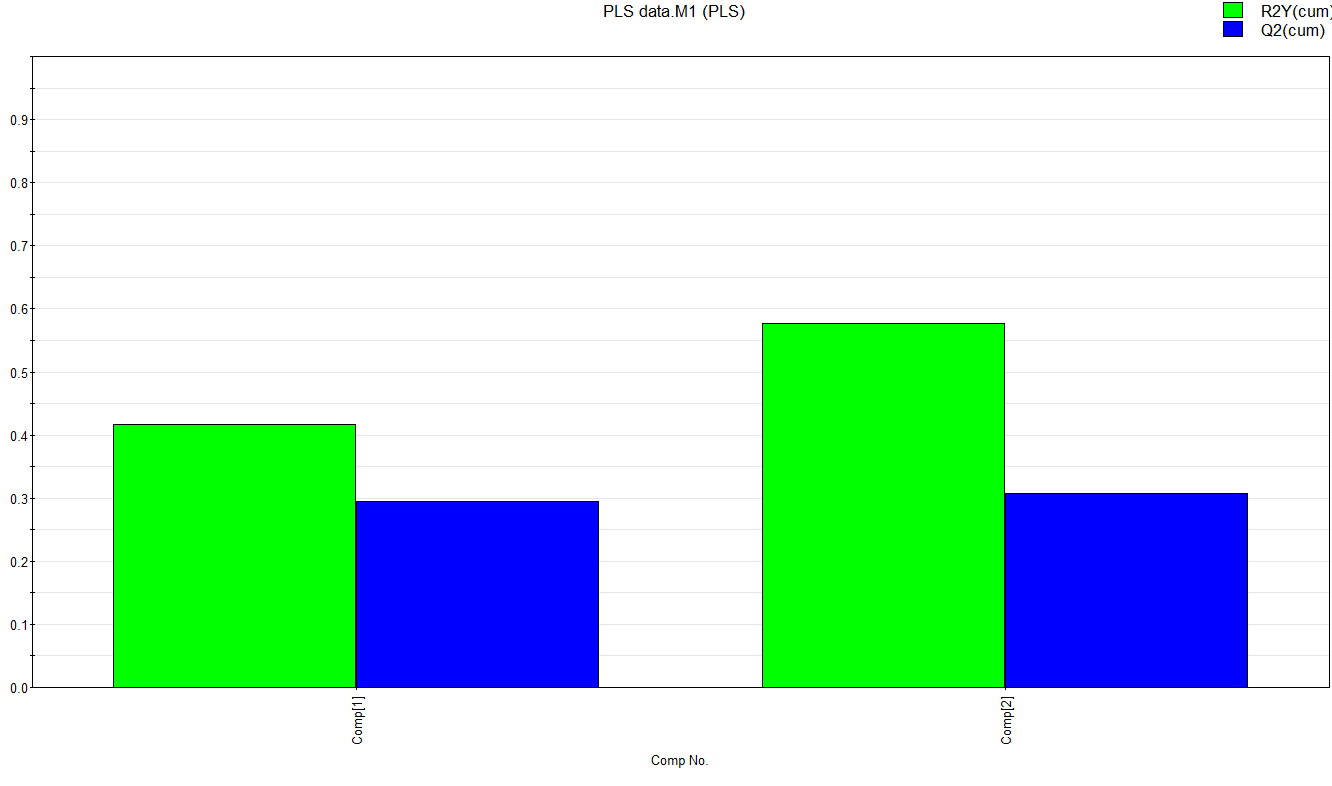


Model overview plot


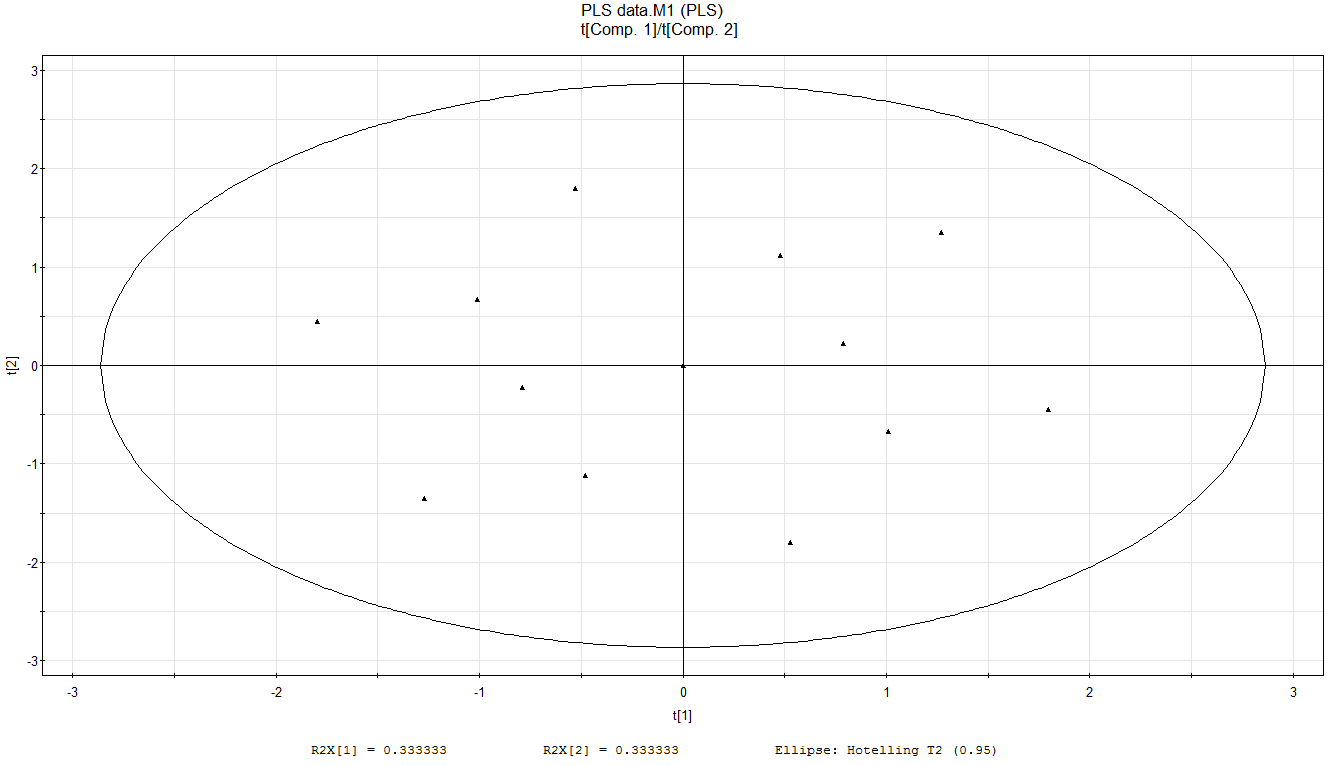


t[1]/t[2] scatter plot


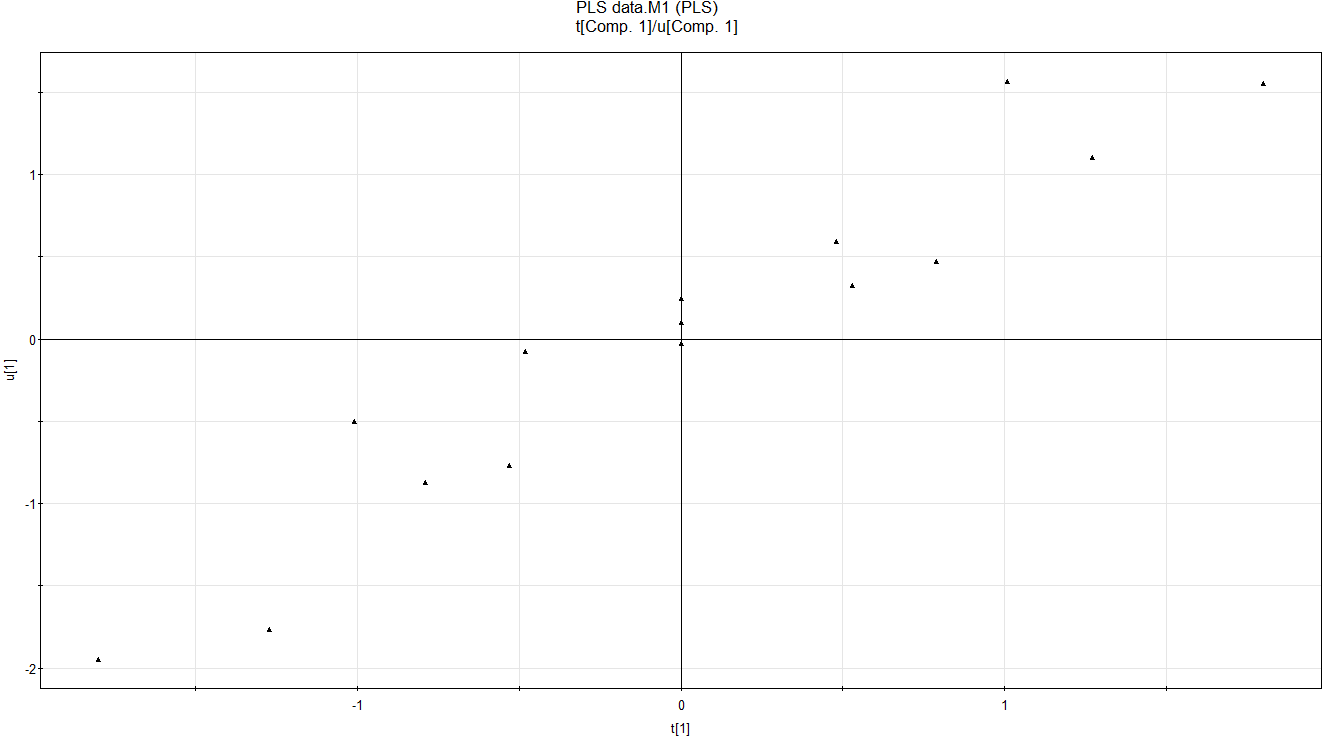


t[1]/u[1] Scatter Plot


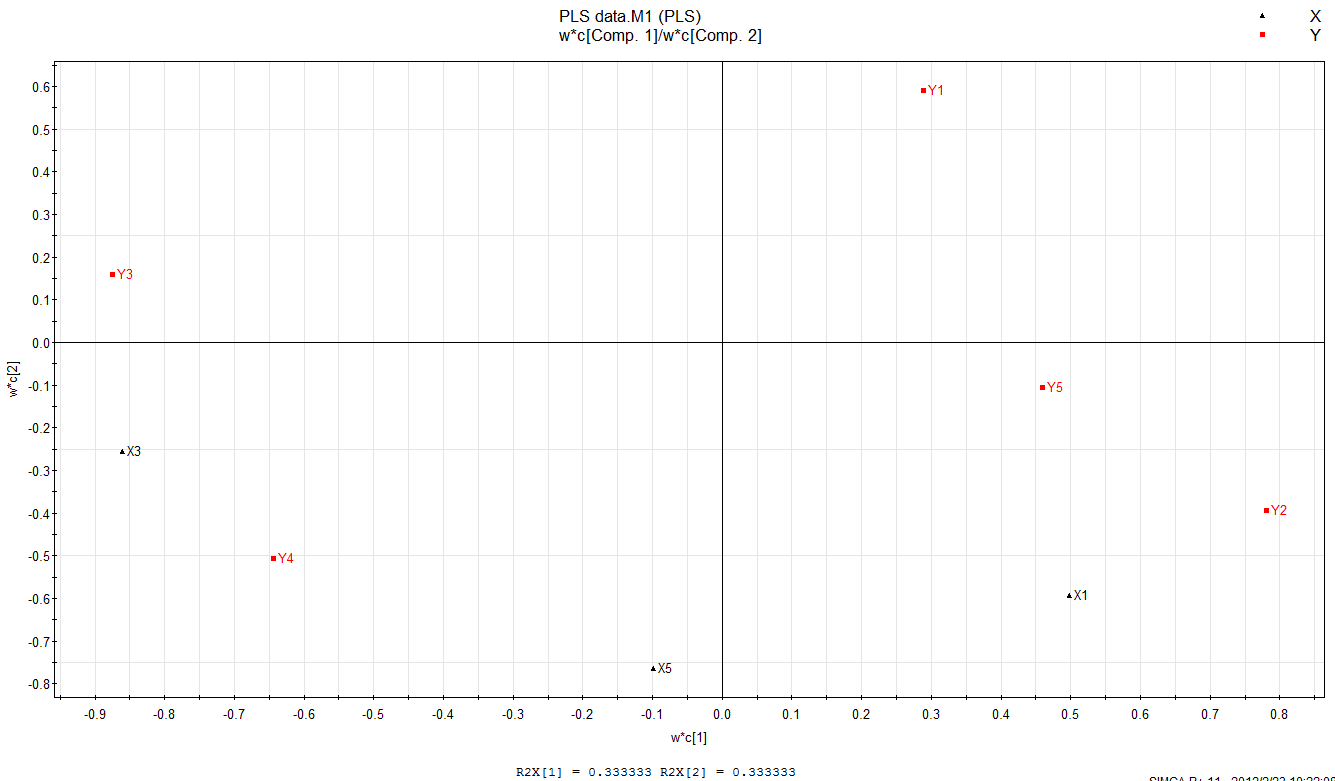


W*c[1]/w*c[2] Scatter Plot
